# A150 THE PARKINSON’S DISEASE-ASSOCIATED MUTATION LRRK2 G2019S INCITES NEUTROPHILIC INFLAMMATION AND INCREASED TISSUE DAMAGE IN A MOUSE MODEL OF INFECTIOUS COLITIS.

**DOI:** 10.1093/jcag/gwae059.150

**Published:** 2025-02-10

**Authors:** N L Oliveira, J Pei, S J Recinto, A Kazanova, C Queiroz-Junior, Z Li, C T Ribeiro, A J Milnerwood, M Desjardins, A Thanabalasuriar, J A Stratton, S Gruenheid

**Affiliations:** Microbiology and Immunology, McGill University, Montreal, QC, Canada; Microbiology and Immunology, McGill University, Montreal, QC, Canada; Microbiology and Immunology, McGill University, Montreal, QC, Canada; Microbiology and Immunology, McGill University, Montreal, QC, Canada; Universidade Federal de Minas Gerais, Belo Horizonte, MG, Brazil; Microbiology and Immunology, McGill University, Montreal, QC, Canada; Microbiology and Immunology, McGill University, Montreal, QC, Canada; Microbiology and Immunology, McGill University, Montreal, QC, Canada; Universite de Montreal, Montreal, QC, Canada; Microbiology and Immunology, McGill University, Montreal, QC, Canada; Microbiology and Immunology, McGill University, Montreal, QC, Canada; Microbiology and Immunology, McGill University, Montreal, QC, Canada

## Abstract

**Background:**

The gut-brain axis is an area of intense interest in Parkinson’s disease (PD) research. PD is characterized by inflammation and gastrointestinal (GI) dysfunction, such as recurrent constipation, dysphagia, and intestinal bacterial overgrowth. GI symptoms in PD appear decades before motor symptoms. Mutations in the protein leucine-rich repeat kinase 2 (LRRK2) are associated with increased risk for PD and inflammatory bowel disease. Among them, the LRRK2 Gly2019Ser is the most common PD-associated mutation. It is unknown whether and how the LRRK2 mutation affects intestinal inflammation susceptibility or pathogenesis of PD.

**Aims:**

To understand how the LRRK2 mutation affects intestinal inflammation susceptibility or pathogenesis of PD.

**Methods:**

LRRK2 knock-in G2019S (GKI) and WT mice were infected with the mouse-specific pathogen *Citrobacter rodentium* and intestinal immune response was evaluated by single cell RNA sequencing (scRNAseq), flow cytometry, and ELISA.

**Results:**

Results revealed that, at day 7 of infection, GKI mice had increased histopathology in colon after infection, compared to WT mice. The most prominent features were inflammatory infiltrate, edema, erosion, and goblet cells loss, although the control of infection was comparable, since the CFU counts were equal in both infected groups. Multiplex analysis of colon homogenates demonstrated that LRRK2 mutation changed cytokine production in response to the infection, including increase in G-CSF, CCL5, and decrease of IL-6 and CXCL2 levels. Myeloperoxidase ELISA in colon tissue showed a tendency to be higher in GKI mice, after infection, compared to WT mice. Single cell RNA sequencing (scRNAseq) of colonic LP cells demonstrated that after infection, LRRK2 G2019S mutation has a great impact in the number of differentially expressed genes (DEGs) on innate immune cells, such as neutrophils, monocytes, and NK cells. In fact, neutrophil was the only cell type proportionally significant after infection. Their DEGs were mostly upregulated and accounted for cell signalling, inflammatory pathways, myeloid leukocyte migration, and interferon response. In addition, flow cytometry revealed that after *C. rodentium* infection, GKI mice had increased neutrophil infiltration of colonic lamina propria (LP), compared to WT infected mice. Analysis in CD4 T cells by scRNA sequencing and RT-qPCR showed an increase in IL-17a expression, known for its G-CSF stimulating neutrophil migration effect.

**Conclusions:**

Our results suggest that the LRRK2 GKI mutation promotes neutrophilic pathology in response to intestinal bacterial infection and this could be linked to an increase of IL-17a-mediated immune response.

**Funding Agencies:**

Aligning Science Across Parkinson’s: ASAP - Michael Fox Foundation

